# Over Expressed TKTL1, CIP-2A, and B-MYB Proteins in Uterine Cervix Epithelium Scrapings as Potential Risk Predictive Biomarkers in HR-HPV-Infected LSIL/ASCUS Patients

**DOI:** 10.3389/fonc.2019.00213

**Published:** 2019-04-03

**Authors:** Anna Chiarini, Daisong Liu, Mario Rassu, Ubaldo Armato, Claudio Eccher, Ilaria Dal Prà

**Affiliations:** ^1^Human Histology and Embryology Unit, University of Verona Medical School, Verona, Italy; ^2^Plastic Surgery Department, Xiangya Third Hospital, Central South University, Changsha, China; ^3^Microbiology and Virology Unit, San Bortolo Hospital, Vicenza, Italy; ^4^Villa Bianca Hospital, Trento, Italy

**Keywords:** LSIL, ASCUS, human cervical carcinoma, oncogenic papillomaviruses, TKTL1, CIP-2a, B-MYB, predictive biomarker

## Abstract

High oncogenic risk human papillomaviruses (HR-HPVs) promote cervical carcinoma development, the fourth most common feminine cancer. A slow oncodevelopmental phase—defined histopathologically as Cervical Intraepithelial Neoplasia (CIN) grades 1–3, or cytologically as Low- or High-grade Squamous Intraepithelial Lesions (LSIL or HSIL)—precedes the malignancy. Cervical carcinoma screenings through HR-HPV genotyping and Pap smears are regularly performed in Western countries. Faulty cytology screening or genotyping or patients' non-compliance with follow-ups can let slip an oncoprogression diagnosis. Novel biomarker tests flanking HR-HPV genotyping and cytology could objectively predict the risk of disease progression thus helping triage LSIL/ASCUS patients. Here, anonymized leftovers of fresh cervical epithelium scrapings from twice (LSIL/ASCUS and HR-HPV DNA)-positive and twice (Pap smear- and HR-HPV DNA)-negative (control) patients in a proteome-preserving solution served to assess the biomarker worth of three cervical carcinoma-related proteins, i.e., B-MYB (or MYBL2), Cancerous Inhibitor of PP2A (CIP-2a), and transketolase-like1 (TKTL1). Leftovers anonymity was strictly kept and storage at −80°C, protein extraction, immunoblotting, and band densitometry were blindly performed. Only after tests completion, the anonymous yet code-corresponding HR-HPV-genotyping and cytology data allowed to assign each sample to the twice-positive or twice-negative group. Descriptive statistics showed that the three proteins levels significantly increased in the twice-positive vs. twice-negative scrapings. Diagnostic ROC curve analysis identified each protein's Optimal Decision Threshold (OTD) showing that TKTL1 and CIP-2a are stronger risk predictive biomarkers (Sensitivity, 0.91–0.93; Specificity, 0.77–0.83) than B-MYB. Logistic Regression coupled with Likelihood-Ratio Tests confirmed that a highly significant relation links increasing TKTL1/CIP-2a/B-MYB protein levels in twice-positive cervical scrapings to the risk of HR-HPV-driven oncoprogression. Finally, a 3 year clinical follow-up showed that 13 patients (50% of total) of the twice-positive group with biomarker values over OTDs compliantly underwent scheduled colposcopy and biopsy. Of these, 11 (i.e., 84.7%) received a positive histological diagnosis, i.e., CIN1 (*n* = 5; 38.5%) or CIN2/CIN2+ (*n* = 6; 46,2%). Therefore, TKTL1/CIP-2a/B-MYB protein levels could objectively predict oncoprogression risk in twice (HR-HPV- and Pap smear)-positive women. Further studies will assess the translatability of these findings into clinical settings.

## Introduction

Human papillomavirus (HPV) infections drive ~5% of all human cancers (1). So far, DNA sequence information has identified more than 200 HPV genotypes (2–4). Of these, 51 are sexually transmitted *mucosal* α*-HPV genotypes* which, according to their epidemiological association with cancers of uterine cervix, vagina, vulva, anus, and penis, are gathered in three subtype groups: (i) *high-risk HPVs* (*HR-HPVs*); (ii) *likely HR-HPVs* (*l-HR-HPVs*); and (iii) *low-risk HPVs* (*LR-HPVs*) (5, 6). Additionally, *cutaneous* β*-HPV genotypes*, may cause skin warts and cancers (7–10). An acute infection due to HR o LR-HPVs spontaneously heals within 2–3 years in ~90% of the patients. However, concurring co-factors (e.g., smoking, oral contraceptives, pregnancy, Herpes virus type 2 infections, *etc*.) may promote a persisting HR-HPV integration into the infected cells' genome triggering an oncogenic progression in the remaining ~10% of cases (11–14). Notably, human cervical carcinoma is the fourth most common female cancer worldwide (11, 15). These malignancies are generally preceded by a pre-invasive or *in situ* disease, defined (i) via histopathological criteria *as Cervical Intraepithelial Neoplasia* (CIN), distinguished in three sequential grades, i.e., *CIN1, CIN2*, and *CIN3*; and (ii) via cytological criteria as *Low-grade-* or *High-grade Squamous Intraepithelial Lesions* (*LSIL* or *HSIL*, respectively) or as *Atypical Squamous Lesions of Undetermined Significance (ASCUS)* (14, 16). Typically, a fraction of the women diagnosed with LSIL or ASCUS lesions may have an underlying CIN grade histopathology or a cervical carcinoma (17, 18). The best management options for such LSIL or ASCUS women are controversial (19, 20). Moreover, a discrete fraction of non-compliant patients drops from any proposed follow-up program (21, 22). Hence, although it should be the first choice when patient's compliance is uncertain, direct colposcopy referral for all LSIL/ASCUS cases would mostly detect only minor dysplasia (23). Conversely, a negative cytological misdiagnosis would delay proper treatment (24). Then again, a negative HR-HPV DNA test might be more falsely reassuring than a negative cytological test (25). Finally, present genotyping does not cover oncogenic l-HR- and LR-HPV subtypes.

Each Western country addresses this somewhat murky diagnostic situation differently. The Italian Association of Cervical Screening Programs (GISCI) binding guidelines emphasize the need for using first the HR-HPV genotyping followed, if positive, by the cytology test and again, if positive, by colposcopy; negative women should be retested after 1–3 years (26, 27). However, it would be both scientifically and clinically helpful to rely also upon specific biomarkers *objectively* predicting the actual risk of progression for any cytological precancerous lesion in HR-HPV DNA-positive and Pap smear-positive patients (22, 28).

Therefore, the present work aimed at assessing the potential worth of three proteins—i.e., B-MYB (or MYBL2), Cancerous Inhibitor of PP2A (CIP-2a), and transketolase-like1 (TKTL1)—as risk predictive biomarkers of disease progression. We chose these proteins because they are over expressed in advanced dysplastic lesions and cervical carcinomas [(13, 29–35); see the Discussion for more details]. We used as starting materials, under strictly anonymized conditions, the leftovers of cervical epithelium scrapings from HR-HPV DNA-positive and LSIL/ASCUS diagnosed (i.e., *twice-positive*) patients and, as controls (Ctr), scraped leftovers from HPV DNA-negative and Pap smear-negative (i.e., *twice-negative*) patients. Our herein reported biochemical results integrated by the results of a subsequent 3-year follow-up of the compliant fraction (50%) of the same twice-positive patients indicate that increases in TKTL1, CIP-2a, and B-MYB proteins above their respective Optimal Decision Threshold (ODT) values may robustly predict the risk of an active HR-HPV-driven oncogenesis.

## Materials and Methods

### Samples

Following the suggestions of European Union guidelines, population-based organized cervical screening programs have been ongoing since 1998 in the Veneto and Trentino-Alto Adige Regions (North-East Italy) (36–38). Every 3 or 5, years women aged 19–64 are invited to undergo Pap smear cytology and HPV-DNA genotyping at no personal expense. The screening procedures are organized and overseen by the Local Healthcare Units. The screening protocol follows the GISCI guidelines (26) and a Health Technology Assessment Report on HPV DNA-based primary screening (27). Besides the official letter of invitation, the eligible women receive an a leaflet with information about HPVs, the HR-HPV test, and the screening program. If women do not act in response to the first invitation, they receive a second informing reminder by mail. Women coming of their own accord to the Hospital test site sign the informed consent prior to the sampling of their cervical cells. The collection of the anonymized leftover samples of human cervical epithelial cells took place in Veneto and Trentino Hospitals. Given the particular study type ethical review and approval was not required as per local legislation. Just after sampling, the cervical leftovers were dipped into a freezing solution (90%^v/v^ fetal bovine serum (FBS) + 10%^v/v^ DMSO), coded to anonymize them, and next stored at −80°C until brought to our lab where they received the second coding. Preliminary investigations (not detailed here) led to choose the FBS+DMSO freezing solution as it preserved the integrity of the cervical cells' proteome. Several other commercial solutions evaluated in parallel, which contained organic and fixative solvents (e.g., Thin Prep^TM^ *Hologic USA*, Cytofast^TM^ *Hospitex International Italy, etc*.), could not preserve the cells' proteome integrity. HR-HPV DNA genotyping and Pap smear cytology tests were carried out on portions of the original scrapings having the same code as their leftover counterparts at the laboratory services of the Hospitals involved. However, information about the age, HR-HPV DNA genotype(s), and Pap smear diagnosis was kept strictly secret until all the leftovers had undergone the protein immunoblotting tests and their densitometric evaluations. Leftovers with an inadequate protein amount were directly cast out. Leftovers whose HR-HPV DNA genotype could not be retrieved were also disposed of. Leftovers with coexisting infectious conditions other than HR-HPVs were too left out. However, the data from six pilot samples with a cytological diagnosis of inflammation but with a negative result of a complete TVAG (Trichomonas, Candida, Bacterial vaginosis, Chlamydia, and Mycoplasma) test were kept apart as specificity controls. Eventually, 16 both PAP smear-negative and HR-HPV-DNA-negative [“*twice-negative*” or *control* (CTR)] samples, and 26 LSIL/ASCUS and HR-HPV-DNA positive (“*twice-positive*”) samples constituted the main populations studied. Their mean age was for both groups was 34.5 years with a range from 16 to 56 years. Cytological diagnoses proportions were: LSIL, 2/3 and ASCUS, 1/3 of the total cases. LSIL and ASCUS data were pooled together because of the limited numbers of the latter. Moreover, ASCUS HPV-DNA-positive patients were categorized together with LSIL ones because GISCI's guideline indicates that the ASCUS diagnostic category, being *borderline*, must be avoided or limited to a minimum, and the relative samples classified as clearly as possible as LSIL or negative (26, 27). No samples from patients diagnosed as positive for the HR-HPV molecular test and HSIL or ASC-H were obtained as they were directly referred to colposcopy.

### Samples Lysis

This procedure and the subsequent Western immunoblotting was carried out on the anonymized leftover samples blindly, i.e., with no knowledge of their cytological and HR-HPV DNA characteristics. Frozen leftover samples were fast thawed at 45°C and next spun at 200 × *g* for 10 min at 4°C. The resulting pellets were resuspended in 1.0 ml of phosphate buffer saline (PBS) added with complete EDTA–free protease inhibitor cocktail (Roche, Milan). The cells' morphological integrity was checked under an inverted phase-contrast light microscope (IM35, Zeiss). Next, after three washings with PBS + anti-proteolytic cocktail, cell pellets were homogenized in T–PER™ tissue protein extraction reagent (Thermo Scientific, Rockford, USA) also added with the complete EDTA–free protease inhibitor cocktail. Finally, to achieve total cell lysis samples were subjected to one freeze/thaw cycle at −20°C and ice-homogenized with an UltraTurrax™. The protein contents of the extracts were assayed according to Chiarini et al. (39, 40) using bovine serum albumin (BSA) as standard.

### Western Immunoblotting (WB)

WB was performed as detailed in Chiarini et al. (39, 40). Equal amounts of protein lysates (15 μg for the analysis of TKTL1 and CIP-2a and 20 μg for B-MYB) were heat–denatured for 10-min at 70°C in an appropriate volume of 1X NuPAGE LDS Sample Buffer supplemented with 1X NuPAGE Reducing Agent (Life Technologies, Italia). In parallel, 10 μg of protein lysate obtained from cervical carcinoma C4-I cells (39, 40) served as the positive control (PC). The amount of sample to be loaded was determined for each target protein to produce a linear signal response for both the target protein and the loading control (LC). The samples were next loaded on NuPAGE Novex 4–12% Bis–Tris polyacrylamide gel (Life Technologies Italia). After electrophoresis in NuPAGE MOPS SDS Running Buffers using the Xcell SureLock™ Mini–Cell (Life Technologies Italia) (50 min runtime at 200 V constant), proteins were blotted onto nitrocellulose membranes (0.2 μm) by means of iBlot^TM^ Dry Blotting System (Life Technologies Italia). The membranes were probed with the following rabbit polyclonal primary antibodies: (a) anti-TKTL1 (ab 155662, Abcam, UK), diluted 1:1000 in TBST/1% BSA; (b) anti-B-MYB (ab 191064, Abcam), at the concentration of 0.5 μg ml^−1^ in TBST/1% BSA; (c) anti-CIP-2a (ab 99518, ABCAM) diluted 1:1000 in TBST/1% BSA; (d) anti-β actin mouse monoclonal antibody (Santa Cruz Biotechnology) which served as loading control (LC) and was used at 1.0 μg ml^−1^ in TBST/1% BSA. After overnight incubation at 4°C, the membranes were probed with the secondary, alkaline phosphatase conjugated, donkey anti-rabbit IgG (Abcam), diluted 1:3000 in TBST or goat anti-mouse IgG (Thermo) diluted 1:1000 in TBST. BCIP/NBT (cat. # 72091, Sigma) was used as chromogenic blue substrate to detect alkaline phosphatase activity. The membranes were incubated in the substrate solution for 10-min until color development; thereafter, the reaction was stopped by washing the membranes in several changes of bidistilled water. The membranes were scanned in an Odissey™ (LI-COR, Inc. USA) using the 700 channel. A digital image of each *western* blot was saved in TIFF format. The densitometric integrated intensity values of the specific bands for the B-MYB, CIP-2a, and TKTL1 antigens and of the loading controls (LC) were evaluated by means of the Image Studio™ Software, version 5.2, developed by LI-COR Inc. for WB analysis. The densitometric analysis of the specific protein bands pertaining to each sample was performed on three independent immunoblots and the results for each tested protein were presented as the mean of signal intensities ± SEM. To ease calculations all densitometric values were multiplied by E4. In detail, the “*Add Rectangle*” tool in Image Studio Software served to draw a rectangular-shaped box around the bands of interest to quantify the target proteins. The “*Median background method*” allowed to compute the median pixels intensity at the border contouring the band's shape and to subtract the background from the shape intensity. Finally, the “*Signal value*” for each shape was calculated by determining its “*Total signal*” and by subtracting from the latter the product of the “*Background*” multiplied for the “*Area*.”

### Human Cervical Carcinoma C4-I Cells

C4-I cells were plated in 175 cm^2^ plastic flasks (Sarstedt S.r.l., Verona, Italy), and incubated at 37°C in 95%^v/v^ air/5%^v/v^ CO_2_ in a complete medium consisting of 95%^v/v^ Dulbecco's modified Eagle's Minimum Essential Medium (DMEM; Sigma-Aldrich, Milan, Italy), 5%^v/v^ heat-inactivated (56°C for 30 min) FBS (Lonza AG, Basel, Switzerland), and gentamycin (0.1 mg ml^−1^; Lonza). Before reaching confluence, cultures were split at a ratio of 1:6 after briefly incubating them at 18 ± 2°C with 0.025% (w/v) trypsin (Sigma-Aldrich). Protein extracts from the cells of six culture flasks were processed as previously detailed (39, 40) and served as positive controls for B-MYB, CIP-2a, and TKTL1 proteins.

### Cytology and HR-HPV-DNA Genotyping

Pap smear tests were performed on all the cervical epithelium specimens from which the leftovers derived in the laboratories of the involved Hospitals. In parallel, the same scrapings were partly dipped into Thin Prep^TM^ (Hologic USA) to analyze the genotypes of the infecting HPVs using the INFINITY HPV QUAD^TM^ assay developed by AutoGenomics Inc, (Vista, CA, USA), which detects HR-HPV types 16, 18, 31, 33, 35, 39, 45, 51, 52, 56, 58, 59, and 68 (41) or COBAS HPV test (Roche Molecular Diagnostics, Pleasanton, CA, USA) which detects HR-HPV types 16, 18, 31, 33, 35, 39, 45, 51, 52, 56, 58, 59, 66, and 68. Only after completion of immunoblotting and band densitometric intensity assessments on all the leftover samples and prior to be sent to the Verona lab, the results of the cytological and HR-HPV genotyping and the ages of patients were anonymized and each labeled with the same code as the corresponding leftovers. The pairing of the data with the same code allowed their later assignment to the twice-positive or twice-negative group to carry out the statistical work-up. Therefore, no breach of sample anonymity whatsoever took place during all such procedures.

### Statistical Analysis

Descriptive and diagnostic statistical analyses were carried out using the *Analyse-it*™ software package (www.analyse-it.com). Shapiro-Wilk's test revealed that a normal distribution could not be assumed for most of the data groups. Welch's *t*-test or Wilcoxon rank *Z* test (42) were used for the descriptive statistical comparisons of the densitometric integrated intensity results concerning each protein's specific immunoblot bands from twice-negative controls vs. twice (LSIL/ASCUS and HPV DNA)-positive groups. For diagnostic statistics a prevalence of HPV infection of 0.31 was assumed by averaging observations from whole Italy (43, 44). The performance of diagnostic tests, i.e., their ability to correctly identify positive and negative cases over a range of medical decision points, was assessed using non-parametric empirical receiver-operating characteristic (ROC) curve analysis (45). The values ± SEMs of the areas under each ROC curve (AUC) distinguished every sample distribution vs. the no discrimination or chance area, the latter having a value of 0.5 (46). Differences between AUC pairs were assessed via DeLong's non-parametric Z test (47). Youden's J index (or Youden's statistic), which optimizes biomarker performance when equal weight is allotted to Sensitivity and Specificity, was calculated by the non-parametric method of Delong et al. (48–50). The maximum J index value indicated the Optimal Decision Thresholds (ODTs) of the densitometric integrated intensity values for each of the three proteins studied at values of: Sensitivity, 0.933–0.909; and Specificity, 0.833–0.769 (51). Finally, binary Logistic Regression was used to independently confirm that a continuous predictor or explanatory variable did indeed impact on a dependent binary or dichotomous variable (52–56). The tests used were two-sided wherever possible and the level of statistical significance was a *P*-value < 0.05. For more details on statistical methods used see the online Supplemental Material.

## Results

The present work aimed at assessing the worth of three proteins, i.e., B-MYB, CIP-2a, and TKTL1, as risk predictive biomarkers of HR-HPV-driven precancerous disease progression. Anonymized fresh cervical epithelium scrapings leftovers which were “*twice-positive*” having a cytological diagnosis of LSIL/ASCUS and being also HR-HPV DNA-positive for 14 different oncogenic subtypes (see Table 1 for details in this regard) comprised the test group. As observed also by others (57), multiple HR-HPV subtypes infections, with HPV-16, -51, -52, and -56 as the more prevalent ones, occurred in 58% of the leftovers (Table 1). “*Twice-negative*” (both Pap test-negative and HR-HPV DNA-negative) specimens served as controls. To fully preserve the protein complement of the leftovers an *ad hoc* freezing medium (90%^v/v^ FBS + 10%^v/v^ DMSO) was devised and thoroughly tested prior to starting the study. The results proved its clear superiority over any other standard commercial solutions containing organic agents or fixatives, e.g., Thin Prep^TM^ (Hologic, USA), Cytofast^TM^ (Hospitex International, Italy)*, etc*., which were also tested in parallel (not shown). All the leftovers were processed blindly, and their belonging to the twice-positive or twice-negative group became known only after immunoblotting procedures and specific band protein immunoblot densitometry evaluations had been carried out for all the specimens involved.

**Table 1:** Prevalence of HR-HPV DNA subtypes in twice-positive^*^ cervical scrapings.

**HR-HPV subtype**	**Total samples**	**Single HR-HPV infection**	**Multiple HR-HPV infections**	**% multiple infections vs. total samples**
*16*	10	7	3	33.3
*51*	6	2	4	12.5
*52*	5		5	10.4
*56*	5		5	10.4
*18*	5	3	2	10.4
*31*	4	2	2	8.4
*66*	3	2	1	6.3
*35*	2	1	1	4.2
*39*	2		2	4.2
*45*	2		2	4.2
*58*	2	1	1	4.2
*33*	1	1		0
*68*	1	1		0

* *Twice (LSIL/ASCUS and HR-HPV DNA)-positive samples*.

**Figure 1:**
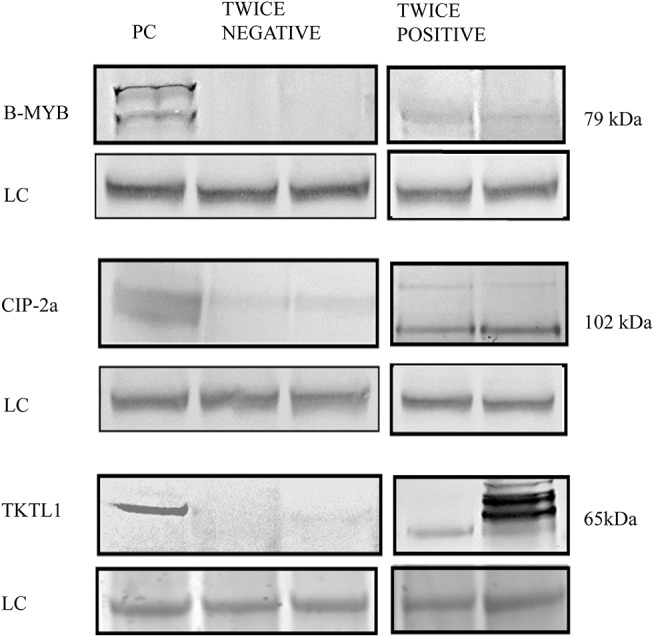
The different expression of putative biomarker proteins B-MYB, CIP-2a, and TKTL1 can be detected in Western immunoblots of cervical epithelium scraping leftovers from twice (LSIL/ASCUS and HR-HPV DNA)-positive and twice (HR-HPV DNA- and Pap-test)-negative patients. The typical immunoblots show also as positive controls (PC) the same proteins as expressed by pre-metastatic human cervical carcinoma C4-I cells. The loading control (LC) protein is β-actin. The samples were run on SDS-PAGE and blotted as detailed in the *Materials and Methods* section.

Figure 1 shows typical immunoblot bands of the three putative biomarker proteins studied. The protein bands from twice (Pap test- and HR-HPV)-negative (Ctr) samples are flanked by corresponding ones from twice (LSIL/ASCUS and HR-HPV)-positive samples. Typical matching proteins bands from pre-metastatic human C4-I cervical carcinoma cells (39, 40) are also displayed as positive controls (PCs).

**Figure 2:**
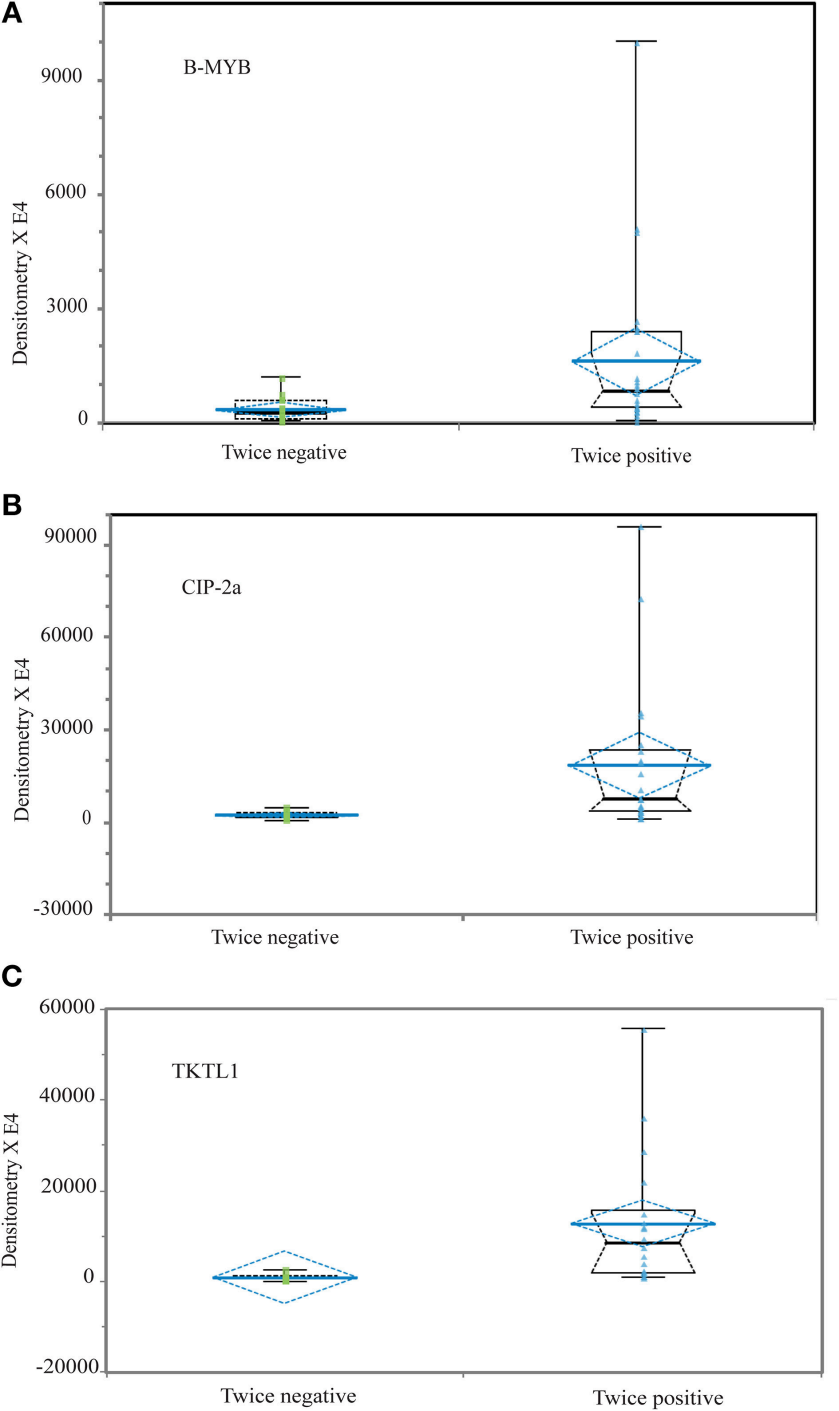
The different expression of putative biomarker proteins B-MYB **(A)**, CIP-2a **(B)**, and TKTL1 **(C)** is significantly increased in twice (LSIL/ASCUS and HR-HPV DNA)-positive fresh cervical epithelium scraping leftovers vs. twice (normal Pap test/HPV DNA-negative, i.e., controls)-negative samples. The data are shown as side by side univariate plots of each protein specific band densitometry values as skeletal notched box charts including from the first to the third quartiles, with the median indicated by a black transverse line, and the minimum and maximum values as whiskers with end caps. The box notch indicates the 95% confidence interval (CI) of the median. A blue line enclosed in a blue dashed diamond indicates the mean value ± SEM. According to Wilcoxon's Z test, the levels of statistical significance of twice-positive vs. twice-negative (control) samples are: B-MYB (*top panel*), *P* = 0.0023; CIP-2a (*middle panel*), *P* < 0.0004; and TKTL1 (*bottom panel*), *P* < 0.0001 (see Table 2).

In absolute terms, the densitometric integrated intensity values of B-MYB immunoblot bands were the lowest while those of CIP-2a the highest ones. In relative terms, the immunoblot bands mean densitometric values were significantly higher—B-MYB, 4.7-fold (*P* = 0.0023); CIP-2a, 7.7-fold (*P* < 0.0001); and TKTL1's 14.6-fold (*P* < 0.0001)—in the twice (LSIL/ASCUS and HR-HPV)-positive samples than in their twice-negative (control) counterparts (Figure 2; Table 2). Therefore, HR-HPV-infection drove a significant overexpression of the three proteins with intensities decreasing in the order TKTL1 > CIP-2a > B-MYB which could be detected through WB and densitometry evaluations in the LSIL/ASCUS cervical epithelium scrapings.

**Table 2:** Densitometric integrated intensity values (xE4) of specific protein bands.

**Putative biomarker**	Twice-negative group (CTR)^*^	Twice-positive group^**^	**% Change vs. CTR**	**P vs. CTR**
B-MYB	344.7 ± 82.5 (*n* = 16)	1609.2 ± 428.7 (*n* = 26)	+366.8	=0.0023
CIP-2a	2,387.5 ± 332.1 (*n* = 16)	18,384.6 ± 5,139.3 (*n* = 26)	+670.0	<0.0001
TKTL1	874.7 ± 166.0 (*n* = 16)	12,786.7 ± 3,454.8 (*n* = 26)	+1361.8	<0.0001

* *CTR, controls*.

** *LSIL/ASCUS and HR-HPV DNA-positive samples*.

**Figure 3:**
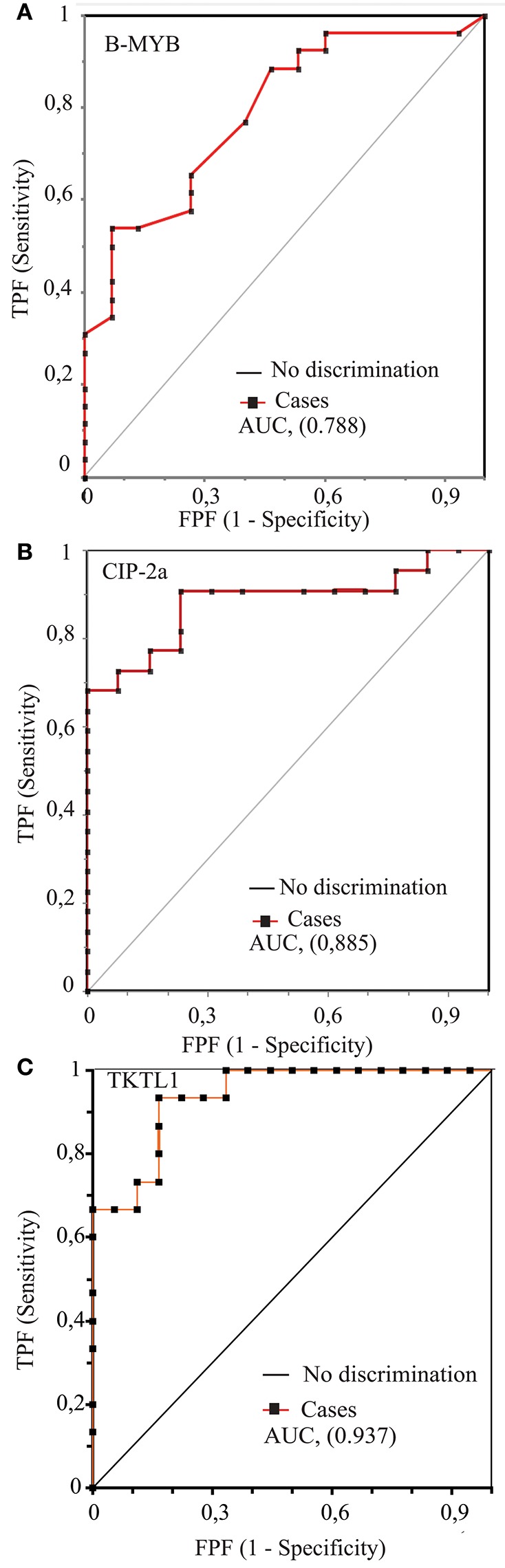
The receiver operating characteristic (ROC) curve analysis of the putative biomarker proteins B-MYB **(A)**, CIP-2a **(B)**, and TKTL1 **(C)**. Details concerning the several diagnostic parameters reckoned from each ROC curve are reported in Table 3 (q. v.). In all three panels the value of the area under the curve (AUC) is shown between brackets as well as the no discrimination (chance) line joining the bottom left angle with the top right angle, the area of which is 0.5. In all the three instances the AUC values significantly differ (*P* < 0.0001) from the no discrimination (chance) area value (see also Table 3). TPF, true positive fraction or Sensitivity. FPF, false positive fraction (1-Specificity).

Receiver operating characteristics (ROC) curves plot the diagnostic accuracy of a putative biomarker across the whole range of potential decision thresholds (45, 46). Figure 3 displays the ROC curves pertaining to each of the three proteins studied. In each instance, the mean value of the area under the respective ROC curve (AUC) (47) significantly (*P* < 0.0001) differed from the value (= 0.5) of the no discrimination or chance area (Figure 3; Table 3). Moreover, the three proteins' AUCs significantly (*P* < 0.001) differed from each other, and both TKTL1's and CIP-2a's AUC values were significantly (*P* < 0.001) higher than B-MYB's. This was the first indication that TKTL1 and CIP-2a likely are more robust biomarkers of HR-HPV-driven risk of disease progression than B-MYB in the twice-positive cervical epithelium scrapings.

**Table 3:** Diagnostic statistic parameter values for each putative biomarker protein.

**Putative biomarker**	**B-MYB**	**CIP-2a**	**TKTL1**
ROC curve AUC ± SEM	0.788 ± 0.0724	0.885 ± 0.0578	0.937 ± 0.0383
AUC 95% CI	0.647–0.930	0.771–0.998	0.862–1.012
AUC DeLong Z statistics	3.99	6.66	11.4
AUC *P* vs. chance^*^	<0.0001	<0.0001	<0.0001
Youden's J index	0.472	0.678	0.833
Optimal decision threshold (ODT)^**^	800	2,380	1,720
% Accuracy at ODT	68.5	85.7	87.9
Sensitivity at ODT	0.913	0.909	0.933
Specificity at ODT	0.538	0.769	0.833
FP proportion at ODT	0.462	0.231	0.167
FN proportion at ODT	0.067	0.091	0.067
Likelihood ratio (+) at ODT	2.02	3.94	5.60
Likelihood ratio (-) at ODT	0.12	0.12	0.08
Predictive value (+) at ODT	0.48	0.64	0.78
Predictive value (-) at ODT	0.95	0.95	0.97
Odds Ratio value at ODT	16.333	33.333	70.000
Cost at ODT	0.189	0.140	0.103

* *Chance area = 0.5*.

** *ODT, values of specific protein band densitometric integrated intensity (xE4)*.

**Figure 4:**
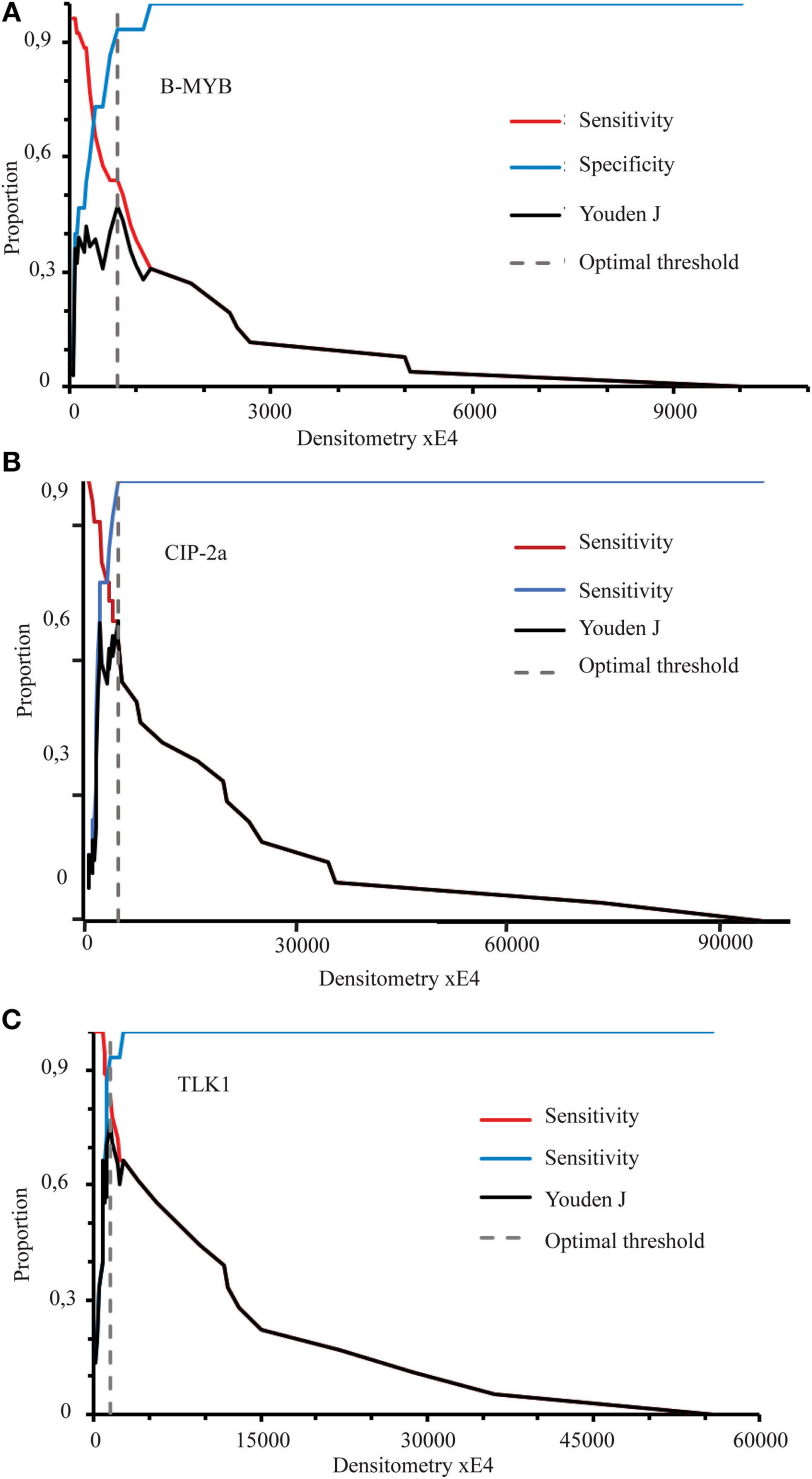
The concurrent changes in the proportion values of Sensitivity, Specificity, and Youden's J index (Youden's statistic) in relation to increasing densitometric integrated intensity values (xE4) of each specific protein band **(A-C)** examined. Optimal Decision Thresholds (ODTs) of the densitometric intensity values for each putative biomarker tested are indicated by vertical dashed lines and coincide with the highest value of the corresponding Jouden's J index.

Precision statistical analysis showed how the values of ROC curve Sensitivity and Specificity and Youden's J index (or Youden's statistic) concomitantly changed for each of the proteins tested along with increasing densitometric values of specific protein bands (Figures 4A–C). Notably, Youden's J index peak value embodies a criterion to choose the Optimum Decision Threshold (ODT)—in this case the specific protein band densitometric integrated intensity values (x E4)—of a dichotomous (0-to-1) diagnostic numeric test (i.e., normal or active oncoprogression) (48, 49).

Remarkably, B-MYB's Youden's J index peak value, *i.e.*, 0.472 (at 0.913 Specificity, 0.538 Sensitivity, 68.5% Accuracy, with a Likelihood Ratio (+) of 2.02, a Predictive Value (+) of 0.48, and a Cost of 0.189) moved to the third rank B-MYB's candidacy as a standalone clinically useful biomarker of an active HR-HPV-driven oncogenesis to be looked for in cervical epithelium scrapings of twice (LSIL/ASCUS and HR-HPV)-positive patients.

Conversely, CIP-2a's peak value of Youden's J index was 0.678 (at 0.909 Sensitivity, 0.769 Specificity, 85.7% Accuracy, with a Likelihood Ratio (+) of 3.94, Predictive Value (+) of 0.64, and Cost of 1.40), which put CIP-2a's biomarker candidacy in the second rank.

Hence, TKTL1 occupied the first rank as candidate biomarker since it exhibited a Youden's J index peak value of 0.833 (at 0.933 Sensitivity, 0.833 Specificity, 87.9% Accuracy, with a Likelihood Ratio (+) of 5.60, Predictive Value (+) of 0.97, and Cost of 0.103) (Table 3). Moreover, the suitability of TKTL1 and CIP-2a as useful clinical biomarkers was strengthened by their very high Odd Ratio values (i.e., the measures of the association between a risk factor and a disease) (48, 49), i.e., CIP-2a, 33.333; and TKTL1, 70.000. In keeping with this, the ODTs (51) of the specific band densitometric integrated intensity values (xE4) were: *CIP-2a*, 2380 with a false positive (FP) proportion of 0.231 and a false negative (FN) proportion of 0.091; and *TKTL1*, 1720 with a FP proportion of 0.167 and a FN proportion of 0.067 (Figure 4 Middle and Bottom Panels; Table 3). Thus, the ODT values of TKTL1 and CIP-2a gained from uterine cervical epithelium scrapings could have a direct clinical significance as they would allow to pinpoint the twice-positive patients with a significant risk of active oncoprogression.

**Figure 5:**
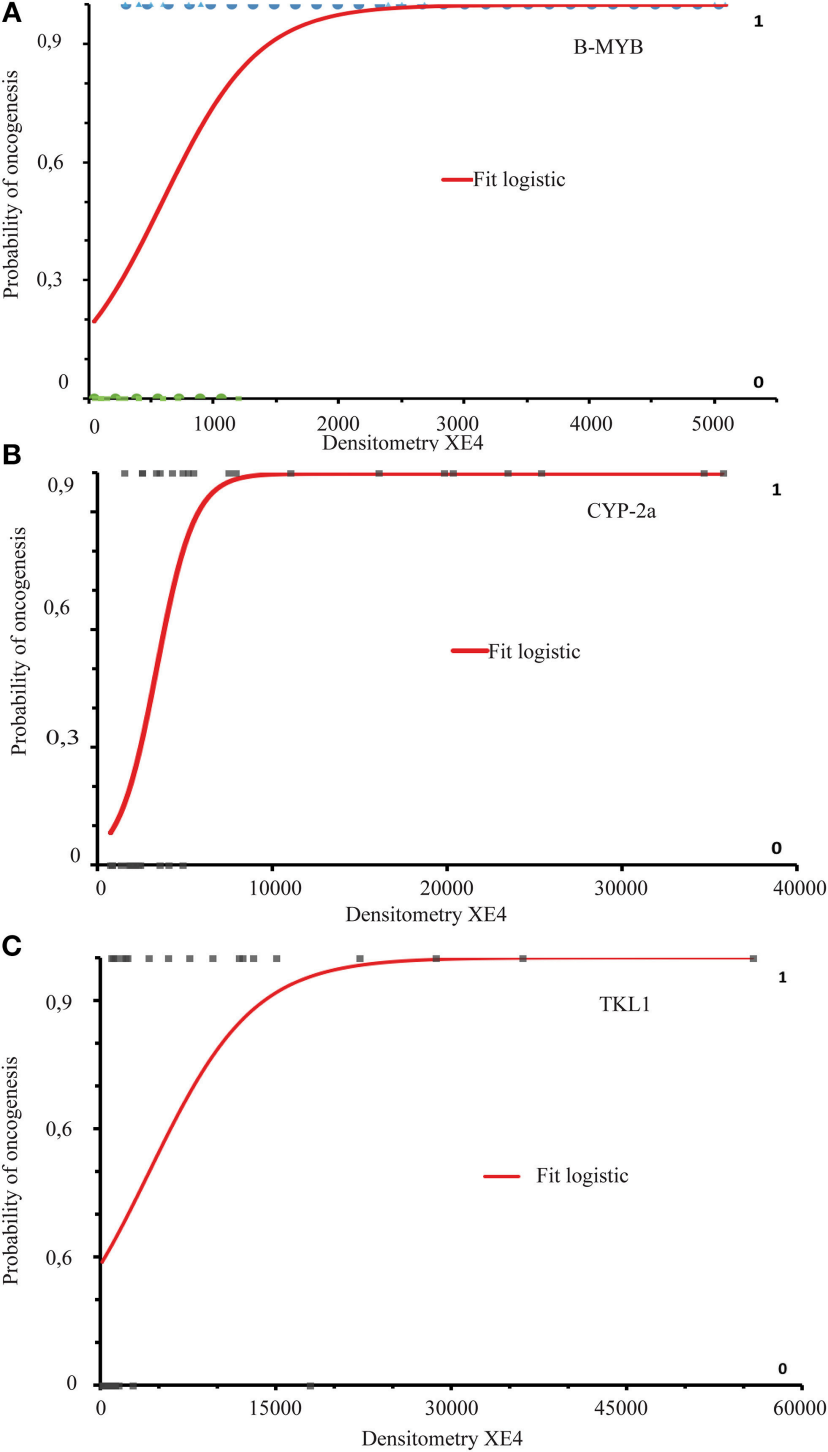
The three binary Logistic Regression curves reach convergence fitting the double-negative (or control) densitometric integrated intensity values with the double (LSIL/ASCUS and HR-HPV DNA)-positive values for each specific protein tested **(A-C)**. The curves show the relationship in terms of Log Odd ratios between a categorical dependent outcome variable (“0” or normal vs. “1” or active oncogenesis) and the continuously increasing values of the predictor or independent variable, that is the specific band densitometry values of each protein studied. The binary Logistic Regression parameters (or coefficient) estimates of the three curves are reported in Table 4. The results of the Likelihood Ratio Tests (LRT or Effect of Model) (not shown here: see Table 5 for details) indicate a statistically significant impact of the increasing independent predictor (i.e., densitometry values) values on the dependent outcome “1” (i.e., ongoing HPV-driven oncogenesis).

An assessment of how the probability of HR-HPV-driven active oncogenesis changed with increasing densitometric values of B-MYB, CIP-2a, and TKTL1 in the cervical epithelium scrapings and its statistical level of significance was carried out via Logistic Regression (54) combined with Likelihood Ratio Test (LTRs or Effect of model) (55). Figure 5 displays the respective fitted Logistic Regression curves indicating the Log Odds ratio (the Logit) of the probability of the oncogenic outcome in relation to increasing densitometric values of each protein in the cervical leftovers examined. Table 4 reports the respective β_0_ and β_1_*x* Logistic Regression parameters estimates, with their corresponding 95% CIs, and SEs.

**Table 4:** Logistic Regression parameters estimates.

**Biomarker**	**Parameter**	**Estimate**	**95% confidence interval (CI)**	**SE**
B-MYB	β_0_	−0.7578	−1.898 to −0.3820	0.58157
	β_1_*x*	0.002177	1.066 to 0.00424	1.0565E-03
CIP-2a	β_0_	−3.073	−5.491 to −0.6554	1.2336
	β_1_*x*	9.226E-04	1.763E-04 to 0.01669	3.8073E-04
TKTL1	β_0_	−0.9249	−1.916 to −0.06620	0.50565
	β_1_*x*	2.234E-04	2.137E-05 to 4.255E-04	1.0309E-04

The LTR tests demonstrate that in all the three protein instances, the Log Likelihood (or the Deviance from the theoretical best fit) of the Full Model with all the β_0_+β_1_*x* predictor data was significantly smaller than the Log Likelihood of the Null Model with only the β_0_ intercept and no β_0_+β_1_*x* predictor data (Table 5). Therefore, the predictor significantly improved the Model fit, meaning that the increasing densitometric values of the three proteins tested in scraped cervical epithelium samples of patients with an ASCUS/LSIL cytology diagnosis could significantly impact on the outcome of active HR-HPV-driven oncogenesis. Hence, though having different degrees of clinical translatability, B-MYB, CIP-2a, and TKTL1 are biomarkers of HR-HPV-driven risk of disease progression which can be detected in cervical epithelium scrapings with a LSIL/ASCUS cytological diagnosis.

**Table 5:** Likelihood Ratio Test (LRT or Effect of model).

**Biomarker**	**Source**	Log likelihood^**^	DF^*^	**G^**2**^ statistic**	***P***
**B-MYB**	*Difference*	5.6899	1	11.38	0.0007
	*Full model*	21.235	39		
	*Null model*	26.925	40		
**CIP-2a**	*Difference*	10.388	1	20.78	<0.0001
	*Full model*	12.106	31		
	*Null model*	22.494	32		
**TKTL1**	*Difference*	5.8293	1	11.66	0.0006
	*Full model*	17.679	32		
	*Null model*	23.508	33		

* *DF, degrees of freedom*.

** *, a measure of Deviance from optimal fit (see text)*.

Finally, a pilot study involving six HR-HPV DNA-negative cervical epithelial scrapings with a generic cytological diagnosis of inflammation but with a negative result of the complete TVAG (Trichomonas, Candida, Bacterial vaginosis, Chlamydia, and Mycoplasma) test showed that no significant difference could be detected in the expression of TKTL1, CIP-2a, and B-MYB proteins and in ROC curve and Logistic Regression/LRT diagnostic parameters vs. 16 double-negative control samples (data not shown). Though preliminary, these findings further strengthen the specificity of TKTL1 and CIP-2a as robust biomarkers of HR-HPV-driven active oncogenesis detectable in cervical LSIL/ASCUS scrapings.

### Clinical Three-Year Follow-Up Results

A total of 13 patients (50%) of twice-positive patients, *whose TKTL, CIP-2A, and B-MYB markers values were above corresponding OTDs* (Figure 4 Middle and Bottom Panels; Table 3), compliantly underwent a scheduled enrollment colposcopy coupled with cervical biopsy during the following three years in order to discriminate benign from precancerous lesions. In this group, 11 women (84.6%) received a positive histological diagnosis: the most frequent histological upshot was CIN1 (*n* = 5; 38.5% of total cases), followed by CIN2+ (*n* = 4; 30.8% of total cases) and by CIN2 (*n* = 2; 15.4% of total cases). These follow-up histology results strengthen the view that the biomarkers we analyzed in the cervical scrapings are endowed with a risk predictive value of disease progression.

## Discussion

The sexually transmitted oncogenic HPVs are a real bane of human life. It is estimated that in the USA from 28 to 46% of the women under 25 years have been or are infected with HPVs; novel infections are ~6.2 million per year; chronic HPV carriers are ~20 million; histopathology reveals that ~330.000 women present a high-grade (CIN 2/3) cervical dysplasia, and ~1.4 million a low-grade (CIN 1) one; and ~3,700 women die of cervical carcinoma each year; of course, worldwide data have a much greater impact (4, 58). Presently, cervical carcinoma-preventive screenings are regularly performed every 2–3 years in Western countries. Notably, HR-HPV-16 and -18 subtype infections cause 71% of all the cervical carcinomas (11). This figure rises to 90% when HPV-6, -11, -16, -18, -31, -33, -45, -52, and -58 infections are brought in (58). Reportedly, multiple HPV types are found in low-grade cervical lesions, but in high-grade lesions HPV-16 and -18 types prevail (28, 57, 59). As data in Table 1 show, HP-16, -51, -52, -56, and -18 were in decreasing order the most prevalent subtypes in twice-positive cervical epithelium samples, and the identification of multiple infecting HPV DNA subtypes in the same specimen occurred in 58.3% of the samples.

Most important, HR-HPVs can drive the development of cancers in both sexes at the level of vulva, anus, penis, prostate, oropharynx, skin, and neck, particularly in HIV-infected patients (28, 60–62). Moreover, HPV infections' social impact is striking due to (i) emotional reactions of the patients and family members aroused by the awareness of the HR-HPVs infection and by the reiteration of follow-up screenings; and to (ii) growing health service diagnostic and care costs (63). In HR- or LR-HPV-infected women, cytological screening identifies the presence of LSIL or HSIL or ASCUS lesions (14, 16, 64–66). The triage of LSIL/ASCUS-diagnosed patients is problematic because not only of the huge numbers involved but also of the lack of generally accepted management protocols. In addition, follow-up patients may let slip their tests; and faulty cytology screening results or incomplete or false-negative HR-HPV genotyping (for LR-HPV subtypes no commercial diagnostic kits are available) may fail to spot patients with a HPV-driven oncogenic progression (17, 19–22, 67). To solve this diagnostic problem, a proper and fast triage of ASCUS/LSIL cervical lesions particularly in young women would require a third leg, i.e., the assay of a novel biomarker(s) objectively predicting the risk of an active HR-HPV-driven disease and might pinpoint the patients needing a quick colposcopy referral besides being clinically useful worldwide including the underdeveloped countries (4, 11, 62).

In this regard, several biomarker quests have been undertaken. Co-detection of p16 and Ki67, two well-known surrogate biomarkers of cell-cycle de-regulation, was initially suggested to unmask HPV-induced oncogenic transformation (68, 69). More recently, Nuovo et al. (70) identified as putative biomarkers importin-β, exportin-5, Mcl1, and cFlip, the expression of which is raised in CIN 1/2 histopathology lesions. In turn, Markovic et al. (71) reported as a useful metabolic biomarker the expression of Cervical Acid Phosphatase (CAP) by epithelial cells in Pap smears which antecedes suspicious morphological changes. Gomih et al. (72) showed that an increased methylation of *IGF2AS* and *PEG10* gene regions associates with a chance of CIN 1 → CIN 2 progression. Also, Bhatia et al. (73) suggested a chemokine pattern (i.e., CCL2, CCL3, CCL4, CXCL1, CXCL8, and CXCL12) assessed in cervical liquid-based cytology samples as a biomarker revealing precancerous cervical lesions. Moreover, Jin et al. (74) reported that the combined enzyme–linked immunosorbent detection of Sialyl Lewis A and HPV-16 L1 improved the ability to distinguish CIN 1, CIN 2, CIN 3 grades and cancer groups patients from normal subjects. Most recently, Jin et al. (74, 75) looked for (i) autoantibodies directed against Cancer Antigens 19-9 (CA19-9) and 15-3 (CA15-3), carcinoembryonic antigen (CEA), c-Myc, p53, heat shock proteins (Hsp)27 and Hsp70, in the sera of HPV-infected patients; and (ii) for six tumor-associated proteins, of which combinations of Sialyl Lewis A and p53 would discriminate cancer from normal condition in exfoliated cervical cells. Finally, Li et al. (76) suggested to detect CIN 2+ stages via *PAX 1* gene methylation analysis in the triage of ASCUS/LSIL cases.

We independently devised to directly test the worth as biomarkers of three proteins as HPV-oncogenesis risk beacons in cervical epithelium scrapings from “*twice-positive*” patients, that is having a LSIL/ASCUS cytological diagnosis and a demonstrated and identified HR-HPV-DNA genotype infection as compared to “*twice-negative*,” that is both Pap smear test-negative and HR-HPV DNA-negative patients. The expression of the three putative protein biomarkers we evaluated, i.e., B-MYB, CIP-2A, and TKTL1, was known to increase in histopathology sections of CIN2+ and of cervical carcinoma cones (29–32).

In more detail, B-MYB is a DNA-binding transcription factor, the expression and transcriptional activity of which increase in cells progressing from G1 phase to S phase (or DNA synthesis) of the mitotic cycle. Immunohistochemically, being weakly expressed B-MYB is hard to detect in most normal cervical epithelial cells. Conversely, B-MYB is over expressed and localizes in the cell nuclei of CIN 2+, cervical glandular intraepithelial neoplasia, and invasive cervical carcinoma specimens (13, 29, 33). B-MYB associates with the HPV E7 protein and interacts with phosphorylated retinoblastoma protein (pRb), a tumor suppressor protein, and with p107, a pRb-related protein (13).

In turn, CIP-2a promotes tumorigenesis and, in general, tumor progression by inhibiting the activity of protein phosphatase 2A (PP2A)—a prerequisite for the oncogenic transformation of human cells—and by stabilizing c-MYC in different tumors. CIP-2A is overexpressed in cervical carcinomas as it positively correlates with HPV-16 E6 and E7 proteins expression in CIN3 and cervical cancer tissues and cells (33). CIP-2a expedites the G1/S transition by modulating Cdk1 and Cdk2 activities in a B-MYB–dependent manner. Indeed, B-MYB might be the downstream target of CIP-2a, since CIP-2a silencing inhibited the expression of B-MYB in human cervical SiHa cancer cells (34, 35). CIP-2A identification in HPV-associated cancers implies the clinical prominence of this protein as a cancer biomarker and a potential therapeutic target (30).

Finally, TKTL1 is a key enzyme in the pentose phosphate pathway, which plays as a crucial role in the progression of cervical neoplasia (31). TKTL1 expression increases in CIN 2/3 or frank neoplasia stages and in neoplastic HeLa cells (32).

The present findings prove that our method of preserving cervical epithelium scraping leftovers to obtain from them integral protein lysates brings about results agreeing with histopathological data reported in the Literature (29–35). The procedures we used are simple and the results highly reproducible. Hence, TKTL1, CIP-2a, and B-MYB testing could be fully automatized using an *ad hoc* equipment and software (this is a current undertaking of our lab). Arguably, such automated procedures could be used in mobile labs crisscrossing underdeveloped countries to screen for HR-HPV-infected women at impending risk of cervical carcinoma.

Previously, the majority cervical carcinoma-related biomarkers were discovered in histological sections of cervical tissue cone biopsies taken at colposcopy. Although such biomarkers may increase the accuracy of the histological diagnosis of cancer, histopathology cannot be the primary methodological approach in cervical carcinoma screenings. This would be impractical as it could hardly deal with large numbers of LSIL/ASCUS bioptic samples particularly in underdeveloped countries. Conversely, using the very same cervical epithelium scrapings to perform standard cytology, HR-HPV DNA genotyping, and biomarker(s), such as TKTL1 and/or CIP-2a expression levels analysis via standard protein lysates Western immunoblotting and specific band densitometry is a feasible undertaking.

In keeping with the just mentioned Literature, our present findings reveal for the first time that it is feasible to detect an increased expression of TKTL1, CIP-2A, and B-MYB in the cervical epithelium scrapings of HR-HPV DNA-positive women having a LSIL/ASCUS cytological diagnosis. Notwithstanding B-MYB's weaker expression, its statistically significant diagnostic indexes make it a still potentially translatable risk predictive biomarker of HR-HPV-driven disease progression. B-MYB's “weakness” may be due to its role as a transcription factor (33), which does not require its synthesis in huge amounts. Moreover, B-MYB's near absence in twice-negative (control) scraping samples is due to its declining synthesis in the terminally differentiating cells of the higher layers of the cervical epithelium (33).

Strengths of this works are: the novel diagnostic approach using cervical epithelium scraping leftovers; feasible and practical methodology used for protein expression analysis; use of biomarkers of the cervical epithelium as objective indicators of HR-HPV-driven disease; the precise assessment of the ODT values for each biomarker which reduces to a minimum the size of false-positive and false-negative findings; and the matching of the biomarker data with follow up data in 85% of the compliant patients.

Limitations of this work are: grouping of ASCUS and LSIL patients in one category because, according to Italian GISCI directive, LSIL or negative diagnoses have mostly supplanted the ASCUS one; the relatively small numbers of patients examined which warrants further studies and follow-ups with larger cohorts.

In conclusion, our biochemical, statistical and three-year follow up findings indicate TKTL1, CIP-2A, and B-MYB as potential robust risk predictive biomarkers of an active onco-developmental process driven by HR-HPV subtypes.

## Data Availability

The raw data supporting the conclusions of this manuscript will be made available by the authors, without undue reservation, to any qualified researcher.

## Ethics Statement

We considered our study exempt from the above ethics requirements because (1) the anonymized cervical epithelium scrapings leftovers we used for our study would have been otherwise totally destroyed and scientifically wasted; (2) we could strictly maintain the complete anonymity of the same scrapings leftovers along all the procedural steps of our research: no subject can be identified from our data; (3) upon our preliminary verbal query Vicenza's Hospital Ethics Committee members answered that there was no need to submit a research protocol for approval since we would have been using anonymized leftovers and keeping them anonymous; and (4) FDA's regulations which exempt the use of leftovers from ethics requirements are taken as reference indications by Hospital Ethics Committees of Veneto and Trentino (Italy).

## Author Contributions

AC, DL, and ID equally contributed to design the study and its methodology, to carry out the laboratory work, to collect and interpret the data, and to draft the manuscript. UA and MR supervised the technological aspects of the project and contributed to the interpretation of the data. MR and CE assisted in the clinical supervision and sample collection. UA and CE performed the statistical descriptive and diagnostic analyses of the data and wrote substantial parts of the manuscript. UA revised the manuscript draft. All authors read and approved the manuscript in its present final form.

### Conflict of Interest Statement

The authors declare that the research was conducted in the absence of any commercial or financial relationships that could be construed as a potential conflict of interest.

## Abbreviations

ASCUS: atypical squamous lesions of undetermined significance
AUC: area under the (ROC) curve
95% CI: 95% confidence interval
CIN: cervical intraepithelial neoplasia
CIP-2a: cancerous inhibitor of PP2A
FN: false negative
FP: false positive
GISCI: Italian Association of Cervical Screening Programs
HPV: human papilloma virus
HR-HPV: high-risk HPV
HSIL: high-grade squamous intraepithelial lesion
LR-HPV: low-risk HPV
LRT: likelihood ratio test
LSIL, low-grade squamous intraepithelial lesion*;* ODT: optimal decision threshold
ROC: receiver operator characteristic
TKTL1: transketolase-like1
TNF: true negative fraction (or specificity)
TPF: true positive fraction (or sensitivity).

## References

[1] Schiller JT Lowy DR. Understanding and learning from the success of prophylactic human papillomavirus vaccines. Nat Rev Microbiol. (2012) 10:681–92. 10.1038/nrmicro2872 22961341 PMC6309166

[2] Bernard HU Burk RD Chen Z van Doorslaer K zur Hausen H de Villiers EM. Classification of papillomaviruses (PVs) based on 189 PV types and proposal of taxonomic amendments. Virology. (2010) 401:70–9. 10.1016/j.virol.2010.02.002 20206957 PMC3400342

[3] Antonsson A Forslund O Ekberg H Sterner G Hansson BG. The ubiquity and impressive genomic diversity of human skin papillomaviruses suggest a commensalic nature of these viruses. J Virol. (2000) 74:11636–41. 10.1128/JVI.74.24.11636-11641.2000 11090162 PMC112445

[4] Manini I Montomoli E. Epidemiology and prevention of human papillomavirus. Ann Ig. (2018) 30:28–32. 10.7416/ai.2018.2231 30062377

[5] International Agency for Research on Cancer IARC Monographs on the Evaluation of Carcinogenic Risks to Humans. IARC Monographs Volume 100 (B). Lyon: IARC (2012). Available online at: https://monographs.iarc.fr/wp-content/uploads/2018/06/mono100B-11.pdf

[6] Schiffman M Clifford G Buonaguro FM. Classification of weakly carcinogenic human papillomavirus types: addressing the limits of epidemiology at the borderline. Infect Agent Cancer. (2009) 4:8. 10.1186/1750-9378-4-8 19486508 PMC2694995

[7] zur Hausen H. Viruses in human cancers. Science. (1991) 254:1167–73. 10.1126/science.1659743 1659743

[8] Smola S. Human papillomaviruses and skin cancer. Adv Exp Med Biol. (2014) 810:192–207. 25207367

[9] Patel H Polanco-Echeverry G Segditsas S Volikos E McCart A Lai C. Activation of AKT and nuclear accumulation of wild type TP53 and MDM2 in anal squamous cell carcinoma. Int J Cancer. (2007) 121:2668–73. 10.1002/ijc.23028 17721920

[10] Talora C Cialfi S Oliviero C Palermo R Pascucci M Frati L. Cross talk among Notch3, pre-TCR, and Tal1 in T-cell development and leukemogenesis. Blood. (2006) 107:3313–20. 10.1182/blood-2005-07-2823 16368887

[11] Li N Franceschi S Howell-Jones R Snijders PJ Clifford GM. Human papillomavirus type distribution in 30,848 invasive cervical cancers worldwide: variation by geographical region, histological type and year of publication. Int J Cancer. (2011) 128:927–35. 10.1002/ijc.25396 20473886

[12] Walboomers JM Jacobs MV Manos MM Bosch FX Kummer JA Shah KV. Human papillomavirus is a necessary cause of invasive cervical cancer worldwide. J Pathol. (1999) 189:12–19. 10.1002/(SICI)1096-9896(199909)189:1<12::AID-PATH431>3.0.CO;2-F 10451482

[13] International Agency for Research on Cancer IARC Monographs on the Evaluation of Carcinogenic Risks to Humans, Volume 90. Human Papillomaviruses. Lyon: IARC (2007). Available online at: https://monographs.iarc.fr/wp-content/uploads/2018/06/mono90.pdf

[14] Schiffman M Kjaer SK Chapter 2: natural history of anogenital human papillomavirus infection and neoplasia. J Natl Cancer Inst Monogr. (2003) 2003:14–9 10.1093/oxfordjournals.jncimonographs.a003476 12807940

[15] Ferlay J Soerjomataram I Dikshit R Eser S Mathers C Rebelo M. Cancer incidence and mortality worldwide: sources, methods and major patterns in GLOBOCAN 2012. Int J Cancer. (2015) 136:E359–86. 10.1002/ijc.29210 25220842

[16] Arends MJ Buckley CH Wells M. Aetiology, pathogenesis, and pathology of cervical neoplasia. J Clin Pathol. (1998) 51:96–103. 10.1136/jcp.51.2.96 9602680 PMC500501

[17] Srodon M Parry Dilworth H Ronnett BM. Atypical squamous cells, cannot exclude high-grade squamous intraepithelial lesion: diagnostic performance, human papillomavirus testing, and follow-up results. Cancer. (2006) 108:32–8. 10.1002/cncr.21388 16136595

[18] Davey DD Greenspan DL Kurtycz DF Husain M Austin RM. Atypical squamous cells, cannot exclude high-grade squamous intraepithelial lesion: review of ancillary testing modalities and implications for follow-up. J Low Genit Tract Dis. (2010) 14:206–14. 10.1097/LGT.0b013e3181ca66a6 20592556

[19] Nakamura Y Matsumoto K Satoh T Nishide K Nozue A Shimabukuro K. HPV genotyping for triage of women with abnormal cervical cancer screening results: a multicenter prospective study. Int J Clin Oncol. (2015) 20:974–81. 10.1007/s10147-015-0789-4 25652908

[20] Schiffman M Vaughan LM Raine-Bennett TR Castle PE Katki HA Gage JC. A study of HPV typing for the management of HPV-positive ASC-US cervical cytologic results. Gynecol Oncol. (2015) 138:573–8. 10.1016/j.ygyno.2015.06.040 26148763 PMC4556538

[21] Kupets R Lu Y Vicus D Paszat L Ontario cancer screening research network. Are there flaws in the follow-up of women with low-grade cervical dysplasia in Ontario? J Obstet Gynaecol Can. (2014) 36:892–9. 10.1016/S1701-2163(15)30438-2 25375302

[22] Solares C Velasco J Álvarez-Ruiz E González-Fernández L Encinas AI Astudillo A. Expression of p16/Ki-67 in ASC-US/LSIL or normal cytology with presence of oncogenic HPV DNA. Anticancer Res. (2015) 35:6291–5. 26504065

[23] Kyrgiou M Kalliala I Mitra A Ng KY Raglan O Fotopoulou C. Immediate referral to colposcopy versus cytological surveillance for low-grade cervical cytological abnormalities in the absence of HPV test: a systematic review and a meta-analysis of the literature. Int J Cancer. (2017) 140:216–23. 10.1002/ijc.30419 27603593

[24] Arbyn M Roelens J Simoens C Buntinx F Paraskevaidis E Martin-Hirsch PP Human papillomavirus testing versus repeat cytology for triage of minor cytological cervical lesions. Cochrane Database Syst Rev. (2013) 3:CD008054 10.1002/14651858.CD008054.pub2 PMC6457841 23543559

[25] Koliopoulos G Nyaga VN Santesso N Bryant A Martin-Hirsch PP Mustafa RA. Cytology versus HPV testing for cervical cancer screening in the general population. Cochrane Database Syst Rev. (2017) 8:CD008587. 10.1002/14651858.CD008587.pub2 28796882 PMC6483676

[26] Gruppo Italiano Screening Citologico (GISCi) Raccomandazioni sul Test HR-HPV Come Test di Screening Primario e Rivisitazione del Ruolo del Pap Test. Belluno: Evidenzia (2010). p. 19 Available online at: http://www.gisci.it/documenti/documenti_gisci/documento_hpv.pdf

[27] Ronco G Biggeri A Confortini M Naldoni C Segnan N Sideri M Health Technology Assessment. Ricerca del DNA di papillomavirus umano (HPV) come test primario per lo screening dei precursori del cancro del collo dell'utero. HPV DNA based primary screening for cervical cancer precursors. Epidemiol Prev. (2012) 36(3–4 S1):e1–72. 22828243

[28] Bzhalava D Guan P Franceschi S Dillner J Clifford G. A systematic review of the prevalence of mucosal and cutaneous human papillomavirus types. Virology. (2013) 445:224–31. 10.1016/j.virol.2013.07.015 23928291

[29] Astbury K McEvoy L Brian H Spillane C Sheils O Martin C. MYBL2 (B-MYB) in cervical cancer: putative biomarker. Int J Gynecol Cancer. (2011) 21:206–12. 10.1097/IGC.0b013e318205759f 21270603

[30] Soofiyani SR Hejazi MS Baradaran B. The role of CIP2A in cancer: a review and update. Biomed Pharmacother. (2017) 96:626–33. 10.1016/j.biopha.2017.08.146 29035828

[31] Kohrenhagen N Voelker HU Schmidt M Kapp M Krockenberger M Frambach T. Expression of transketolase-like 1 (TKTL1) and p-Akt correlates with the progression of cervical neoplasia. J Obstet Gynaecol Res. (2008) 34:293–300. 10.1111/j.1447-0756.2008.00749.x 18686341

[32] Chen H Yue JX Yang SH Ding H Zhao RW Zhang S. Overexpression of transketolase-like gene 1 is associated with cell proliferation in uterine cervix cancer. J Exp Clin Cancer Res. (2009) 28:43. 10.1186/1756-9966-28-43 19331662 PMC2669053

[33] Martin CM Astbury K Kehoe L O'Crowley JB O'Toole S O'Leary JJ. The use of MYBL2 as a novel candidate biomarker of cervical cancer. Methods Mol Biol. (2015) 1249:241–51. 10.1007/978-1-4939-2013-6_18 25348311

[34] Liu J Wang X Zhou G Wang H Xiang L Cheng Y. Cancerous inhibitor of protein phosphatase 2A is overexpressed in cervical cancer and upregulated by human papillomavirus 16 E7 oncoprotein. Gynecol Oncol. (2011) 122:430–6. 10.1016/j.ygyno.2011.04.031 21575984

[35] Tian Y Chen H Qiao L Zhang W Zheng J Zhao W. CIP2A facilitates the G1/S cell cycle transition via B-Myb in human papillomavirus 16 oncoprotein E6-expressing cells. J Cell Mol Med. (2018) 22:4150–60. 10.1111/jcmm.13693 29893470 PMC6111863

[36] Zorzi M Del Mistro A Farruggio A de'Bartolomeis L Frayle-Salamanca H Baboci L. Use of a high-risk human papillomavirus DNA test as the primary test in a cervical cancer screening programme: a population-based cohort study. BJOG. (2013) 120:1260–7. 10.1111/1471-0528.12272 23786222

[37] Coleman D Day N Douglas G Farmery E Lynge E Philip J. European guidelines for quality assurance in cervical cancer screening. Europe against cancer programme. Eur J Cancer. (1993) 29A (Suppl 4): S1–S38. 8274301

[38] Arbyn M Anttila A Jordan J Ronco G Schenck U Segnan N. European guidelines for quality assurance in cervical cancer screening. Second edition summary document. Ann Oncol. (2010) 21:448–58. 10.1093/annonc/mdp471 20176693 PMC2826099

[39] Chiarini A Marconi M Pacchiana R Dal Prà I Wu J Armato U. Role-shifting PKCζ fosters its own proapoptotic destruction by complexing with Bcl10 at the nuclear envelope of human cervical carcinoma cells: a proteomic and biochemical study. J Proteome Res. (2012) 11: 3996–4012. 10.1021/pr3000464 22812606

[40] Chiarini A Liu D Armato U Dal Prà I. Bcl10 crucially nucleates the pro-apoptotic complexes comprising PDK1, PKCζ and caspase-3 at the nuclear envelope of etoposide-treated human cervical carcinoma C4-I cells. Int J Mol Med. (2015) 36:845–56. 10.3892/ijmm.2015.2290 26202083

[41] Erali M Pattison DC Wittwer CT Petti CA. Human papillomavirus genotyping using an automated film-based chip array. J Mol Diagn. (2009) 11:439–45. 10.2353/jmoldx.2009.080154 19644025 PMC2729841

[42] Rosner B Glynn RJ Lee ML. The Wilcoxon signed rank test for paired comparisons of clustered data. Biometrics. (2006) 62:185–92. 10.1111/j.1541-0420.2005.00389.x 16542245

[43] Baussano I Franceschi S Gillio-Tos A Carozzi F Confortini M Dalla Palma P. Difference in overall and age-specific prevalence of high-risk human papillomavirus infection in Italy: evidence from NTCC trial. BMC Infect Dis. (2013) 13:238. 10.1186/1471-2334-13-238 23706168 PMC3669053

[44] Chironna M Tafuri S De Robertis AL Sallustio A Morea A Napoli A. Prevalence of HPV infection and genotype distribution in women from Africa seeking asylum in Puglia, Italy. J Immigr Minor Health. (2013) 15:159–63. 10.1007/s10903-012-9698-z 22869450

[45] Hanley JA Mc Neil BJ. The meaning and use of the area under a receiver operating characteristic (ROC) curve. Radiology. (1982) 143:29–36. 10.1148/radiology.143.1.7063747 7063747

[46] Zweig MH Campbell G. Receiver-operating characteristic (ROC) plots: a fundamental evaluation tool in clinical medicine. Clin Chem. (1993) 39:561–77. 8472349

[47] DeLong ER DeLong DM Clarke-Pearson DL. Comparing the areas under two or more correlated receiver operating characteristic curves: a nonparametric approach. Biometrics. (1988) 44:837–45. 10.2307/2531595 3203132

[48] Schisterman EF Perkins NJ Liu A Bondell H. Optimal cut-point and its corresponding Youden Index to discriminate individuals using pooled blood samples. Epidemiology. (2005) 16:73–81. 10.1097/01.ede.0000147512.81966.ba 15613948

[49] Youden WJ. Index for rating diagnostic tests. Cancer. (1950) 3:32–35. 10.1002/1097-0142(1950)3:1<32::AID-CNCR2820030106>3.0.CO;2-3 15405679

[50] Faraggi D The effect of random measurement error on receiving operating characteristic (ROC) curves. Stat Med. (2000) 19:61–70. 10.1002/(SICI)1097-0258(20000115)19:1<61::AID-SIM297>3.0.CO;2-A 10623913

[51] Zou KH Yu CR Liu K Carlsson MO Cabrera J. Optimal thresholds by maximizing or minimizing various metrics via ROC-type analysis. Acad Radiol. (2013) 20:807–15. 10.1016/j.acra.2013.02.004 23582776

[52] Andrade C. Understanding relative risk, odds ratio, and related terms: as simple as it can get. J Clin Psychiatry. (2015) 76:e857–61. 10.4088/JCP.15f10150 26231012

[53] McHugh ML The odds ratio: calculation, usage, and interpretation. Biochem Med. (2009) 19:120–6. 10.11613/BM.2009.011

[54] Hosmer D Lemeshow S Goodness-of-fit test for the multiple logistic regression model. Commun Stat Theory Methods. (1980) 9:1043–69. 10.1080/03610928008827941

[55] Somerville MC Brown RS. Exact likelihood ratio and score confidence intervals for the binomial proportion. Pharm Stat. (2013) 12:120–8. 10.1002/pst.1560 23471686

[56] Allison PD Measures of Fit for Logistic Regression. Statistical Horizons LLC and the University of Pennsylvania. Available online at: https://support.sas.com/resources/papers/proceedings14/1485-2014.pdf

[57] Schmitt M Dondog B Waterboer T Pawlita M Tommasino M Gheit T. Abundance of multiple high-risk human papillomavirus (HPV) infections found in cervical cells analyzed by use of an ultrasensitive HPV genotyping assay. J Clin Microbiol. (2010) 48:143–9. 10.1128/JCM.00991-09 19864475 PMC2812266

[58] de Martel C Plummer M Vignat J Franceschi S. Worldwide burden of cancer attributable to HPV by site, country and HPV type. Int J Cancer. (2017) 141:664–70. 10.1002/ijc.30716 28369882 PMC5520228

[59] Guan P Howell-Jones R Li N Bruni L de Sanjosé S Franceschi S. Human papillomavirus types in 115,789 HPV-positive women: a meta-analysis from cervical infection to cancer. Int J Cancer. (2012) 131:2349–59. 10.1002/ijc.27485 22323075

[60] D'Souza G Gross ND Pai SI Haddad R Anderson KS Rajan S. Oral human papillomavirus (HPV) infection in HPV-positive patients with oropharyngeal cancer and their partners. J Clin Oncol. (2014) 32:2408–15. 10.1200/JCO.2014.55.1341 24778397 PMC4263818

[61] Hoffmann M Quabius ES Tribius S Hebebrand L Görögh T Halec G. Human papillomavirus infection in head and neck cancer: the role of the secretory leukocyte protease inhibitor. Oncol Rep. (2013) 29:1962–8. 10.3892/or.2013.2327 23467841 PMC3658815

[62] Medina-Laabes DT Suarez-Perez EL Guiot HM Muñoz C Colón-López V Tirado-Gómez M. Human papillomavirus correlates with histologic anal high-grade squamous intraepithelial lesions in hispanics with HIV. J Low Genit Tract Dis. (2018) 22:320–5. 10.1097/LGT.0000000000000416 29975333 PMC7375752

[63] Harvey-Knowles JA Kosenko KA. Diagnosing women with HPV: the impact of diagnosis disclosure methods. Patient Educ Couns. (2012) 88:152–6. 10.1016/j.pec.2012.02.002 22370198

[64] Bruno MT Ferrara M Fava V Barrasso G Panella MM. A prospective study of women with ASCUS or LSIL pap smears at baseline and HPV E6/E7 mRNA positive: a 3-year follow-up. Epidemiol Infect. (2018) 146:612–8. 10.1017/S0950268818000250 29465024 PMC9134523

[65] Oliveira GG Oliveira JMDSC Eleutério RMN Barbosa RCC Almeida PRC Eleutério J Jr Atypical squamous cells: cytopathological findings and correlation with HPV genotype and histopathology. Acta Cytol. (2018) 13:1–7. 10.1159/000489386 29898441

[66] Iavazzo C Boutas I Grigoriadis C Vrachnis N Salakos N. Management of ASCUS findings in Papanicolaou smears. A retrospective study. Eur J Gynaecol Oncol. (2012) 33:605–9. 23327054

[67] Massad LS Einstein MH Huh WK Katki HA Kinney WK Schiffman M. 2012 updated consensus guidelines for the management of abnormal cervical cancer screening tests and cancer precursors. J.Low Genit Tract Dis. (2013) 17:S1–S27. 10.1097/LGT.0b013e318287d329 23519301

[68] White C Bakhiet S Bates M Keegan H Pilkington L Ruttle C. Triage of LSIL/ASC-US with p16/Ki-67 dual staining and human papillomavirus testing: a 2-year prospective study. Cytopathology. (2016) 27:269–76. 10.1111/cyt.12317 26932360

[69] Bergeron C Ikenberg H Sideri M Denton K Bogers J Schmidt D. Prospective evaluation of p16/Ki-67 dual-stained cytology for managing women with abnormal Papanicolaou cytology: PALMS study results. Cancer Cytopathol. (2015) 123:373–81. 10.1002/cncy.21542 25891096

[70] Nuovo GJ de Andrade CV Wells SI Brusadelli M Nicol AF. New biomarkers of human papillomavirus infection in acute cervical intraepithelial neoplasia. Ann Diagn Pathol. (2018) 36:21–7. 10.1016/j.anndiagpath.2018.06.008 29966832

[71] Markovic O Markovic N. Cervical acid phosphatase: a biomarker of cervical dysplasia and a potential surrogate endpoint for colposcopy. Dis Markers. (2003-2004) 19:279–86. 10.1155/2004/845798 15258329 PMC3850803

[72] Gomih A Smith JS North KE Hudgens MG Brewster WR Huang Z. DNA methylation of imprinted gene control regions in the regression of low-grade cervical lesions. Int J Cancer. (2018) 143:552–60. 10.1002/ijc.31350 29490428 PMC6019167

[73] Bhatia R Kavanagh K Stewart J Moncur S Serrano I Cong D. Host chemokine signature as a biomarker for the detection of pre-cancerous cervical lesions. Oncotarget. (2018) 9:18548–58. 10.18632/oncotarget.24946 29719625 PMC5915092

[74] Jin Y Kim SC Kim HJ Ju W Kim YH Kim HJ. Use of autoantibodies against tumor-associated antigens as serum biomarkers for primary screening of cervical cancer. Oncotarget. (2017) 8:105425–39. 10.18632/oncotarget.22231 29285261 PMC5739648

[75] Jin Y Kim SC Kim HJ Ju W Kim YH Kim HJ. Use of protein-based biomarkers of exfoliated cervical cells for primary screening of cervical cancer. Arch Pharm Res. (2018) 41:438–49. 10.1007/s12272-018-1015-5 29492827

[76] Li SR Wang ZM Wang YH Wang XB Zhao JQ Xue HB. Value of PAX1 methylation analysis by MS-HRM in the triage of atypical squamous cells of undetermined significance. Asian Pac J Cancer Prev. (2015) 16:5843–6. 10.7314/APJCP.2015.16.14.5843 26320460

