# Supplementary material for: Over Expressed TKTL1, CIP-2A, and B-MYB Proteins in Uterine Cervix Epithelium Scrapings as Potential Risk Predictive Biomarkers in HR-HPV-Infected LSIL/ASCUS Patients
Source: Front Oncol. 2019 Apr 3;9:213. doi: 10.3389/fonc.2019.00213 (PMC6456695; doi:10.3389/fonc.2019.00213)
Supplement: Supplementary file 1 [file Data_Sheet_1.docx]

**Supplemental Materials**

OVER EXPRESSED TKTL1, CIP-2a, AND B-MYB PROTEINS IN UTERINE CERVIX EPITHELIUM SCRAPINGS AS POTENTIAL RISK PREDICTIVE BIOMARKERS IN HR-HPV-INFECTED LSIL/ASCUS PATIENTS

Anna CHIARINI^1*§^, Daisong LIU^1,2*§^, Mario RASSU^3^, Ubaldo ARMATO^1^,

Claudio ECCHER^4^, Ilaria DAL PRÀ^1*§^

^1^*Human Histology & Embryology Unit, University of Verona Medical School, Verona, Venetia, Italy*

^2^*Plastic Surgery Department, Xiangya Third Hospital, Central South University, Changsha, Hunan China*

^3^*Microbiology & Virology Unit, San Bortolo Hospital, Vicenza, Venetia, Italy*

*^4^Villa Bianca Hospital, Trento, Trentino, Italy*

*To whom correspondence should be addressed at: [anchiari@gmail.com](mailto:anchiari@gmail.com), [ippdalpra@gmail.com](mailto:ippdalpra@gmail.com), liudaisong_1987@qq.com

^§^These authors equally contributed to the paper

***Supplemental Statistical Methods***

Shapiro-Wilk’s test revealed that a normal distribution could not be assumed for most of the data groups. Failures of Levene’s test for equality of variances and unequal sizes of sample groups required the use of nonparametric Welch’s *t*-test or Wilcoxon rank Z test (**1S**) for the descriptive statistical comparisons of the densitometric integrated intensity results concerning each protein’s specific immunoblot bands from twice-negative controls *vs.* twice (LSIL/ASCUS and HPV DNA)-positive groups. The descriptive data were graphically represented as skeletal notched box charts including from the first to the third quartiles, with the median indicated by a transverse black line, and the minimum and maximum values as whiskers with end caps; the box notch denoted the 95% confidence interval (CI) of the median; a blue line enclosed in a blue-dashed diamond indicated the mean value ± SEM. For diagnostic statistics a prevalence of HPV infection of 0.31 was assumed by averaging observations from whole Italy (**2S,3S**). The **performance** of diagnostic tests, i.e. their ability to correctly identify positive and negative cases over a range of medical decision points, was assessed using nonparametric empirical receiver-operating characteristic (ROC) curve analysis (**4S**). ROC curves require a dichotomous 0-or-1 scale variable specifying the true state of the subject (e.g. normal or pathological) and a continuous scale diagnostic test (e.g., the densitometric integrated intensity values measured for each of the three putative biomarkers evaluated). To ROC curves were constructed plotting the various true positive (TP) proportions (or Sensitivity) on the abscissa against false positive (FP) proportions (or 1–Specificity) on the ordinate. The values ± SEMs of the areas under each ROC curve (AUC) distinguished every sample distribution *vs.* the no discrimination or chance area, the latter having a value of 0.5 (**5S**). Differences between AUC pairs were assessed via DeLong’s nonparametric Z test (**6S**)**.** Youden’s J index (or Youden’s statistic), which optimizes biomarker performance when equal weight is allotted to Sensitivity and Specificity, was calculated by the nonparametric method of Delong *et al.* (**7S-9S**). The maximum J index value indicated the Optimal Decision Thresholds (ODT) of the densitometric integrated intensity values for each of the three proteins studied at values of: Sensitivity, 0.933-0.909; and Specificity, 0.833-0.769 (**10S)**. ODT values also specified the minimum theoretical healthcare Cost per patient. The following equation allowed to calculate the values of the:

*% Accuracy = (TP + TN / TP +TN + FP + FN) x100*

where TN is the true negative and FN is the false negative value for each protein investigated. ODTs for CIP-2a and TKTL1 had accuracy values of 85.7% and 87.9%, respectively. Odd Ratios were assessed according to Andrada and to Mchugh et al. (**11S,12S**). The values of positive and negative Likelihood Ratios and positive and negative Predictive Values were determined via Analyse-it™ software. Finally, binary Logistic Regression was used to independently confirm that a continuous predictor or explanatory variable (e.g. the densitometric integrated intensity values of each protein) did indeed impact on a dependent binary or dichotomous variable with only two possible outcomes coded as 0 (normal) or 1 (oncoprogression). Thus, the best fitting models of the data were determined via Logistic Regression which generated two parameters, i.e. β_0_ (the *intercept* when predictor variable (e.g. densitometric values) is equal to 0), and β_1_x (the *regression coefficient* multiplied by any value *x* of the predictor) and their SEs. The thus obtained formula predicted the Logit transformation or link function or probability (*p*) of the dependent outcome of interest:

*Logit (*or *Log Odds* or *p)* = *ln (p(x)/1-p(x))* = *e*^β0 + β1x^

(where *ln* is the natural logarithm and base *e* denotes the exponential function). Therefore, each Logistic Regression curve modeled as a continuous variable the Logit (or *ln* of the odds) of the chance of an active HR-HPV-driven oncogenesis (the dependent or 0-or-1 outcome) as a function of the predictor or explanatory or independent variable (i.e. densitometric values of the specific protein investigated). The Odds Ratio corresponded to *e*^β1^ and multiplied by *e*^β1^ the predicted outcome for 1-unit increase of the independent variable *x* (**11S-13S**). Via the Likelihood-Ratio Test (LRT) the Deviance (or the measure of the lack of fit to the data in a Logistic Regression model) was assessed which evaluated whether the *Full (*or *Saturated) Model* with the predictor or independent variable β_1_*x* values fitted the data better than the reference *Null Model* which had only the β_0_ intercept and no predictor value. The statistical significance of the difference between the two *Models* yielded a chi-squared G^2^ test assessing the statistical significance of the contribution of the independent variable or predictor to the outcome (**14S,15S**).

**References**

**1S.**  Rosner B, Glynn RJ, Lee ML. The Wilcoxon signed rank test for paired comparisons of clustered data. *Biometrics* (2006) 62:185-92. doi: 10.1111/j.1541-0420.2005.00389.x

**2S**. Baussano I, Franceschi S, Gillio-Tos A, Carozzi F, Confortini M, Dalla Palma P, et al. Difference in overall and age-specific prevalence of high-risk human papillomavirus infection in Italy: evidence from NTCC trial. *BMC Infect Dis.* (2013) 13:238. doi: 10.1186/1471-2334-13-238

**3S**. Chironna M, Tafuri S, De Robertis AL, Sallustio A, Morea A, Napoli A, et al Prevalence of HPV infection and genotype distribution in women from Africa seeking asylum in Puglia, Italy. *J Immigr Minor Health.* (2013) 15:159-63. doi: 10.1007/s10903-012-9698-z

**4S**. Hanley JA, Mc Neil BJ. The meaning and use of the area under a receiver operating characteristic (ROC) curve. *Radiology* (1982) 143: 29-36.

**5S**. Zweig MH, Campbell G. Receiver-operating characteristic (ROC) plots: A fundamental evaluation tool in Clinical Medicine. *Clin Chem*. (1993) 39:561-77.

**6S**. DeLong ER, DeLong DM, Clarke-Pearson DL. Comparing the areas under two or more correlated receiver operating characteristic curves: a nonparametric approach. *Biometrics* (1988) 44:837-45.

**7S**. Schisterman EF, Perkins NJ, Liu A, Bondell H. Optimal cut-point and its corresponding Youden Index to discriminate individuals using pooled blood samples. Epidemiology (2005) 16:73-81. [doi](https://en.m.wikipedia.org/wiki/Digital_object_identifier):[10.1097/01.ede.0000147512.81966.ba](https://doi.org/10.1097/01.ede.0000147512.81966.ba)

**8S**. [Youden WJ.](https://en.m.wikipedia.org/wiki/William_J._Youden) Index for rating diagnostic tests. *Cancer* (1950) 3:32-35.

**9S**. Faraggi D. The effect of random measurement error on receiving operating characteristic (ROC) curves. *Statistics in Medicine* (2000) 19:61-70.

**10S**. Zou KH, Yu CR, Liu K, Carlsson MO, Cabrera J. Optimal thresholds by maximizing or minimizing various metrics via ROC-type analysis. *Acad Radiol.* (2013) 20:807-15. doi: 10.1016/j.acra.2013.02.004

**11S**. Andrade C. Understanding relative risk, odds ratio, and related terms: as simple as it can get. *J Clin Psychiatry.* (2015) 76:e857-61. doi: 10.4088/JCP.15f10150

**12S**. McHugh ML. The odds ratio: calculation, usage, and interpretation. Biochem Med. (2009) 19:120-6. doi: 10.11613/BM.2009.011

**13S**. Hosmer D, Lemeshow S. Goodness-of-fit test for the multiple logistic regression model. *Communications in Statistics⎯Theory and Methods* (1980) 9:1043–69. doi: 10.1080/03610928008827941

**14S**. Somerville MC, Brown RS. Exact likelihood ratio and score confidence intervals for the binomial proportion. *Pharm Stat.* (2013) 12:120-8. doi: 10.1002/pst.1560

**15S**. Allison PD. Measures of fit for logistic regression ([https://support.sas.com/resources/ papers/proceedings14/1485-2014.pdf](https://support.sas.com/resources/%20papers/proceedings14/1485-2014.pdf)) (PDF) Statistical Horizons LLC and the University of Pennsylvania.
